# Effects of Biological Sex and Stress Exposure on Ventromedial Prefrontal Regulation of Mood-Related Behaviors

**DOI:** 10.3389/fnbeh.2021.737960

**Published:** 2021-08-26

**Authors:** Tyler Wallace, Brent Myers

**Affiliations:** Department of Biomedical Sciences, Colorado State University, Fort Collins, CO, United States

**Keywords:** coping, depression, gonadal hormones, infralimbic cortex, reward, sociability, valence

## Abstract

The ventral portion of the medial prefrontal cortex (vmPFC) regulates mood, sociability, and context-dependent behaviors. Consequently, altered vmPFC activity has been implicated in the biological basis of emotional disorders. Recent methodological advances have greatly enhanced the ability to investigate how specific prefrontal cell populations regulate mood-related behaviors, as well as the impact of long-term stress on vmPFC function. However, emerging preclinical data identify prominent sexual divergence in vmPFC behavioral regulation and stress responsivity. Notably, the rodent infralimbic cortex (IL), a vmPFC subregion critical for anti-depressant action, shows marked functional divergence between males and females. Accordingly, this review examines IL encoding and modulation of mood-related behaviors, including coping style, reward, and sociability, with a focus on sex-based outcomes. We also review how these processes are impacted by prolonged stress exposure. Collectively, the data suggest that chronic stress has sex-specific effects on IL excitatory/inhibitory balance that may account for sex differences in the prevalence and course of mood disorders.

## Introduction

Negative mood states are a feature of numerous psychiatric conditions, including anxiety and depressive disorders. Furthermore, major depression, characterized by sadness, reduced motivation, and anhedonia, is the leading cause of years lived with disability worldwide (Friedrich, 2017). Although females are disproportionally impacted by mood disorders, preclinical studies have historically focused on male neural regulation of depression-related behaviors (Kuehner, 2017). However, recent policy and methodological advances have led to the discovery of significant sex differences in the neurobiology of mood. Here, we examine recent studies exploring sex differences in the prefrontal regulation of coping, reward, and sociability.

Clinical neuroimaging studies associate activity in the ventral medial prefrontal cortex (vmPFC) with depressive disorders, and emotional regulation broadly. The vmPFC is also essential for goal-directed and contextually-appropriate behaviors, mood, and stress responding (Nestler et al., 2002; Krishnan and Nestler, 2008; McKlveen et al., 2015). Further, in studies of males and females vmPFC activity is linked with reward processing and positively correlates with the severity of anhedonia (Keedwell et al., 2005; Green et al., 2019). In particular, the vmPFC subregion Brodmann Area 25 (BA25) has decreased volume in MDD patients across sexes, and is a target for deep brain stimulation in treatment-resistant depression (Drevets et al., 1997, 2008; Crowell et al., 2019; Sankar et al., 2019). A meta-analysis of imaging studies using males and females revealed BA25 is responsive to reward and emotional processing (Beckmann et al., 2009), as well as social exclusion (Vijayakumar et al., 2017). Though these studies indicate BA25 activity associates with depressive disorders, results examining BA25 function in depression are varied. Mixed sex studies have reported both reduced metabolic activity in MDD patients (Drevets et al., 1997), as well as hyperactivity in treatment-resistant depression measured by cerebral blood flow (Mayberg et al., 2005). Yet, both conventional antidepressant treatment and deep brain stimulation reduce BA25 activity (Mayberg et al., 2005, 2013). The heterogeneity of neural populations in BA25 may contribute to these divergent results. BA25 is principally composed of excitatory pyramidal neurons that project throughout limbic and brainstem nuclei, with a smaller but diverse population of inhibitory interneurons (Beckmann et al., 2009). Mounting evidence indicates that changes to the excitation/inhibition balance of the vmPFC relate to depressive symptomology, but the contributions of specific neural populations to behavior are difficult to address clinically (Fogaça and Duman, 2019; McKlveen et al., 2019). Further, although clinical studies commonly include both males and females, few analyze outcomes for sex differences (Vijayakumar et al., 2017). However, recent advances in neurobiology have allowed studies to establish casual roles for specific genetically-defined cell populations for processing and regulating behavior across sexes.

The infralimbic cortex (IL) is the rodent anatomical homolog of primate BA25 and is well-positioned for behavioral regulation based on projections throughout the limbic system (Vertes, 2004; Wood et al., 2018). The IL contains glutamatergic projection neurons with inhibitory interneurons providing local network regulation (McKlveen et al., 2015, 2016; Wood et al., 2018). While pharmacological and lesion studies have linked IL activity with depression-relevant behaviors, advances in cell-type specificity have identified sex-dependent roles in stress, reward, and social processes.

## Negative Valence: Stress Coping

Coping with negatively-valenced stimuli involves coordinated behavioral and physiological responses to address real or perceived stressors. Ultimately, stress exposure initiates a neurohormonal cascade that leads to the synthesis of adrenal glucocorticoids that then provide feedback to the brain at glucocorticoid receptors (GR) and mineralocorticoid receptors (MR) to promote behavioral and physiological adaptation (McKlveen et al., 2013; Myers et al., 2014; Herman et al., 2016). An increasing number of studies have identified a role for the IL in stress coping (Table 1), with similarities and differences between male and female rodents.

**TABLE 1:** Negatively valenced stimuli.

References	Sex	Species	IL Manipulation	Timing	Stressor	Outcome
McKlveen et al., 2013	Male	Spraque-Dawley rats	shRNA GR knockdown	5–6 weeks before testing	FST and restraint stress (RS)	FST immobility **↑** RS corticosterone ↑
Slattery et al., 2011	Male	Spraque-Dawley rats	Muscimol	10 min before testing	FST	Immobility ↓
Scopinho et al., 2010	Male	Wistar rats	CoCl_2_ synaptic blockade	10 min before testing	FST	Immobility **↓**
Pace et al., 2020	Male	Spraque-Dawley rats	siRNA vGluT1 knockdown	6 weeks before testing	FST	Immobility **↓**
Fuchikami et al., 2015	Male	Spraque-Dawley rats	Ketamine and muscimol CaMKII-ChR2 stimulation: 15 ms, 10 Hz, 5 mW	24 h before testing	FST	Ketamine: immobility ↓ Ketamine + muscimol: no change ChR2 stim: immobility ↓
Navarria et al., 2015	Male	Spraque-Dawley rats	Scopolamine and muscimol	24 h before testing	FST	Scopolamine: immobility **↓** Scopolamine + muscimol: no change
Hamani et al., 2010	Male	Spraque-Dawley rats	Electrical stimulation	After first FST and prior to second FST	FST	Immobility **↓**
Shimizu et al., 2018	Male	Spraque-Dawley rats	Electrical stimulation	During behavior	EPM	Open arm time ↑
Warden et al., 2012	Male	Long-Evans rats	CaMKII-ChR2 stimulation: 5 ms, 20 Hz, 10–20 mW	During behavior	FST	mPFC stim: no change mPFC to DRN: immobility **↓** mPFC to habenula: immobility ↑
Myers et al., 2017	Male	Spraque-Dawley rats	siRNA vGluT1 knockdown	6 weeks before testing	RS	ACTH ↑ Corticosterone ↑
Schaeuble et al., 2019	Male	Spraque-Dawley rats	siRNA vGluT1 knockdown	6 weeks before testing	RS	Heart rate ↑ Blood pressure ↑
Wallace et al., 2021	Male and Female	Spraque-Dawley rats	CaMKII-ChR2 stimulation: 5 ms, 10 Hz, 3 mW	During behavior	RS and novel environment (NE)	**Males:** RS: corticosterone and glucose ↓ NE: heart rate and blood pressure ↓ **Females:** RS: glucose ↑ NE: heart rate ↑

*IL effects on coping during acute stressors.*

The IL is acutely stress-responsive, as identified by histological markers of neuronal activation, and expresses both GR and MR in multiple cell types (Granholm et al., 1985; Reul and De Kloet, 1986; Cintra et al., 1994; Cullinan et al., 1995; McKlveen et al., 2013, 2016, 2019). Knockdown of male IL GR expression increases passive coping in the forced swim test (FST) and glucocorticoid responses to acute stress (McKlveen et al., 2013). However, pharmacological manipulations of male IL activity have yielded mixed results on coping behaviors. For instance, IL inactivation via GABA_*A*_ receptor activation reduces FST immobility in males, an antidepressant-associated phenotype (Slattery et al., 2011). Similarly, non-specific synaptic blockade in the male IL reduces passive coping in the FST (Scopinho et al., 2010), as does long-term knockdown of IL glutamatergic output in males (Pace et al., 2020). However, both the NMDA antagonist ketamine and the muscarinic antagonist scopolamine increase IL activity and reduce male FST immobility (Fuchikami et al., 2015; Navarria et al., 2015). Further, deep brain stimulation in male rodents reduces passive coping in the FST (Hamani et al., 2010) and increases open arm time in the elevated plus maze (EPM), an anxiolytic-like phenotype (Shimizu et al., 2018). The mixed outcome of pharmacological interventions highlights the need to determine endogenous neural activity patterns during behavior, as well as more temporally- and genetically-specific modulation of neural activity.

*In vivo* recordings indicate the male IL processes anxiogenic stimuli and acute stressors. IL neural activity, measured by multiunit electrodes, increases in the seconds preceding entry into the open arms of the EPM (Shimizu et al., 2018). More generally, multiunit electrode array recordings of male mPFC neurons at the border between the IL and prelimbic (PL) area have variable activity in response to FST. Although many neurons are inhibited during FST, a large portion have selectivity for immobile vs. mobile periods and the majority of those increase activity during mobile periods (Warden et al., 2012). The heterogeneity of cellular responses underscores the need to examine the contributions of specific IL neural populations. Advances in optogenetics have permitted temporally-precise and cell-type specific modulation of IL activity. Activation of male IL glutamatergic neurons 24 h before testing reduces FST passive coping (Fuchikami et al., 2015), suggesting IL stimulation induces pyramidal neuron plasticity. Although, it remains to be determined how this stimulation may regulate IL efferent activity. Optogenetic modulation also permits synaptic stimulation, which indicates output targets differentially influence behavioral outcomes. For instance, Warden et al. stimulated male mPFC (IL and PL) glutamatergic neurons without affecting FST behavior. However, evoked glutamate release from mPFC terminals in the dorsal raphe increased active coping, while projections to the lateral habenula decreased active coping (Warden et al., 2012). Taken together, these results indicate differing behavioral outcomes from modulating male IL activity, likely relating to the cellular specificity of interventions and/or the differential engagement of output targets.

In line with regulating coping behavior, the IL also mediates physiological responses to acute challenges. Viral-mediated knockdown of male pyramidal neuron vesicular glutamate transporter 1 (vGluT1) reduces glutamate release and increases hypothalamic-pituitary-adrenal (HPA) axis, heart rate, and blood pressure responses to restraint (Myers et al., 2017; Schaeuble et al., 2019). Similarly, optogenetic stimulation of male IL glutamatergic neurons reduces both corticosterone and glucose responses to restraint stress, as well as heart rate and blood pressure responses to a novel environment. In contrast, optogenetic stimulation of female IL glutamate neurons increases glucose responses to restraint and heart rate reactivity to a novel environment (Wallace et al., 2021). Collectively, these data suggest that male IL glutamatergic neurons are both necessary and sufficient to reduce autonomic and neuroendocrine responses to stress, while female IL glutamate neurons facilitate stress reactivity. It remains to be determined what mechanisms account for sex differences in IL function. While IL c-Fos expression following FST is similar in males and females, males have greater activation following acute restraint, suggesting differences in stress reactivity may be stimuli-specific (Sood et al., 2018). Further, ovarian hormones may be involved as lateral ventricle infusion of corticotrophin releasing hormone leads to negative correlations between IL c-Fos expression and grooming behavior in both male and diestrus female rats. However, IL activity positively associates with grooming in proestrus females (Wiersielis et al., 2016). Overall IL neural populations signal distinct aspects of stressors, while male IL glutamatergic neural activity constrains the physiological stress response and bidirectionally regulates coping style depending upon projection sites. In contrast, female IL glutamatergic neural activity facilitates the physiological stress response and divergent IL responses to stressors may relate to ovarian hormone signaling.

## Positive Valence: Reward

The IL has a prominent role in coordinating context-appropriate reward-seeking behaviors (Table 2). Pharmacological inhibition of the male rat IL with combined GABA_*A*_ and GABA_*B*_ agonists reduces inhibitory control in a food reward-seeking task (Capuzzo and Floresco, 2020), as well as extinction and renewal of context-conditioned food reward (Eddy et al., 2016). Moorman and Aston-Jones (2015) used a similar pharmacological approach and found that IL inhibition reduces both lever presses to a reward-associated stimulus and extinction of reward-seeking after the stimulus is no longer paired with reward. Furthermore, male IL multiunit potential recordings found that putative pyramidal neurons heterogeneously respond (significantly increase or decrease activity) to cue-evoked reward-seeking and extinction (Moorman and Aston-Jones, 2015). Additionally, male IL neurons have prolonged firing in response to rewarded but not unrewarded operant responses and IL inhibition increases the latency to reward acquisition (Burgos-Robles et al., 2013). IL pyramidal neuron regulation of midbrain dopamine signaling may be important for effects on reward and motivation. Ferenczi et al. utilized a stable-step function opsin (SSFO) to optogentically increase male IL glutamate neuron excitability and found reduced sucrose preference. In females, the SSFO approach also reduces the rewarding quality of ventral tegmental area stimulation in a real-time place preference assay (Ferenczi et al., 2016), suggesting IL inhibition of dopamine signaling may contribute to anhedonia. In contrast, SSFO-induced increases in the excitability of IL GABAergic/vasoactive intestinal peptide (VIP)-expressing interneurons reduces high-calorie palatable food consumption without impacting food reward motivation or low-calorie chow intake (Newmyer et al., 2019). Overall, these studies indicate that male IL neurons signal multiple aspects of food reward acquisition, including contextually-appropriate reward-seeking and behavioral inhibition. Although, specific IL cell populations likely have opposing effects on hedonic feeding.

**TABLE 2:** Positively valenced stimuli.

References	Sex	Species	IL manipulation	Timing	Reward	Outcome
Eddy et al., 2016	Male	Wistar rats	Baclofen/muscimol	30–45 min before testing	Food pellet	Response in rewarding condition ↓ Response in extinct condition ↑
Capuzzo and Floresco, 2020	Male	Long-Evans rats	Baclofen/muscimol	10 min before testing	Sucrose pellet	Inhibitory trial success ↓
Moorman and Aston-Jones, 2015	Male	Sprague-Dawley rats	Electrical recording Baclofen/muscimol	Immediately prior	Sucrose	Recording: IL neural activity during sucrose acquisition ↑ Baclofen/muscimol: Lever press for reward ↓ Extinction ↓
Anthony Burgos-Robles et al., 2013	Male	Sprague-Dawley rats	Electrical recording Muscimol	30 min before testing	Sucrose pellet	Recording: IL neural activity during sucrose acquisition ↑ Muscimol: Reward collection latency ↑
Ferenczi et al., 2016	Male and Female	**Male:** Sprague-Dawley rats **Female:** Long-Evans TH-ChR2 rats	**Male:** CaMKII-SSFO: continuous, 4× over 6 h testing **Female:** CaMKII-SSFO: continuous	During behavior	**Male:** Sucrose **Female:** Dopamine stimulation	**Males:** Sucrose preference ↓ **Females:** Preference for dopamine stimulation ↓
Newmyer et al., 2019	Male	VIP-Cre transgenic mice	Cre-dependent SSFO: continuous	5 min before testing	Palatable high-calorie diet	Palatable food intake ↓
Hurley and Carelli, 2020	Male and Female	Sprague Dawley rats	IL-NAcSh CamKII-ChR2 stimulation: 5 s, 20 Hz, 10 mW	During behavior	Sucrose and stimulation	**Males but not females:** Aversive taste response ↓ **Both sexes:** Respond for stimulation ↑
Wallace et al., 2021	Male and Female	Spraque-Dawley rats	CaMKII-ChR2 stimulation: 5 ms, 10 Hz, 3 mW	During behavior	Real-time place preference	**Males:** Time in stimulation side ↑ **Females:** No preference

*IL effects on reward behavior.*

Fewer publications have investigated the role of the female rodent IL in reward seeking and motivational behaviors. To date, evidence suggests that the female IL may have more limited involvement in reward processing and positive affect. For instance, optogenetic activation of the glutamatergic IL to nucleus accumbens shell (NAcSh) pathway following conditioned taste aversion reduces aversive taste reactivity in males but not females. However, both sexes lever press for IL-NAcSh stimulation and a prior history of IL-NAcSh stimulation increases sucrose preference in males and females (Hurley and Carelli, 2020). Further, optogenetic stimulation of IL glutamatergic neurons induces a real-time place preference in males without affecting place preference or aversion in females, suggesting a positive valence to IL glutamatergic activity in males but not females (Wallace et al., 2021). These studies collectively indicate that, in males, IL activity is necessary for contextual appraisal during reward acquisition and that glutamatergic activity has positive valence. In contrast, current evidence suggests that female IL glutamatergic activity does not affect place preference or conditioned aversion, although activity in specific projections may be rewarding.

## Social Behavior

Reduced sociability is a common symptom of mood disorders. Additionally, decreased motivation for social interaction further worsens the course of depressive illness (Kupferberg et al., 2016). Consequently, determining how neural circuits encode and regulate social behavior is a critical area for investigation. Growing evidence indicates that male IL neural output regulates the affective and motivational processes that underly social interaction (Table 3), possibly through descending limbic integration (Vertes, 2004; Wood et al., 2018). Indeed, pharmacological inactivation of the IL reduces both the frequency and duration of social play in adolescent male rats (Van Kerkhof et al., 2013). Furthermore, Minami et al. (2017) conducted electrical recordings of IL activity during social behavior and found that male IL neurons increase firing during the termination of social behavior, an effect absent in isolation-reared rats suggesting experience-dependent social encoding. SSFO enhancement of male IL glutamatergic neuron excitability reduces social interaction with a juvenile interactor (Ferenczi et al., 2016). In contrast, acute optogenetic stimulation of male IL glutamate neurons increases conspecific social motivation (Wallace et al., 2021), providing further evidence for contextual factors impacting IL-mediated behaviors. Increasing the excitability of male IL GABAergic VIP interneurons reduces novel social investigation, as well as novel object interactions (Newmyer et al., 2019). Other interneuronal investigations examined GABAergic parvalbumin (PV) neurons in the male and female mouse mPFC and found that PV neural activity increases in both sexes during social interactions compared to novel object interactions, a phenotype missing in the CNTNAP2 knockout autism model. Further, SSFO-increased excitability of PV interneurons rescues social deficits in the CNTNAP2 model, without impacting sociability in wildtype mice (Selimbeyoglu et al., 2017). Ultimately, these studies highlight the need to further investigate how specific interneuronal populations within the mPFC differentially encode and modulate social behavior.

**TABLE 3:** Social behavior.

References	Sex	Species	IL manipulation	Timing	Test	Outcome
Van Kerkhof et al., 2013	Male	Wistar rats	Baclofen/muscimol	5 min before testing	Free interaction	Social play ↓
Minami et al., 2017	Adolescent Male	Spraque-Dawley rats	Electrical recordings	During behavior	Free interaction	IL neural activity during interaction termination ↑
Ferenczi et al., 2016	Male	Sprague-Dawley rats	CaMKII-SSFO: continuous, 4× during testing	2 days prior and on testing day	Male juveline in homecage	Social interaction ↓
Selimbeyoglu et al., 2017	Male and Female	PV-Cre C57BL/6J mice	Border of IL and PL GCaMP6f photometry	During behavior	Free interaction	**Both sexes:** PV neural activity during social interaction ↑
Newmyer et al., 2019	Male	VIP-Cre transgenic mice	Cre-dependent SSFO: continuous	5 min before testing	Conspecific in homecage	Social interaction ↓
Wallace et al., 2021	Male and Female	Spraque-Dawley rats	CaMKII-ChR2 stimulation: 5 ms, 10 Hz, 3 mW	During behavior	3-chambered social test	**Males:** Social motivation ↑ **Females:** No change
Huang et al., 2020	Female	C57BL/6J mice	Cre-dependent inhibitory DREADD hM4Di in IL CAV2-Cre in BLA	CNO 30 min before testing	3-chambered social test	Social preference ↓
Pereira and Morrell, 2020	Female	Sprague Dawley rats	Bupivacaine hydrochloride	5 min before testing	Pup-associated conditioned preference	Time in pup-associated zone ↓ Pup retrieval ↓

*IL effects on social behavior.*

Currently, evidence suggests the female IL may have a different role in social behavior. Optogenetic stimulation of IL glutamatergic neurons does not alter female social motivation or social novelty prefrence (Wallace et al., 2021). Further, the female IL appears to be less responsive to social interaction with conspecifics as female rats have less c-Fos expression compared to males after social interaction (Mikosz et al., 2015). Moreover, male rats have greater c-Fos responses to a previously-stressed interactor than an unstressed conspecific, an effect that does not occur in females. This sex difference may be independent of ovarian hormones as both intact cycling females and ovariectomized (OVX) females have similar IL c-Fos following social interaction (Mikosz et al., 2015). However, specific projection-defined IL neurons are necessary for social motivation. Huang et al. (2020) chemogenetically inactivated female basolateral amygdala-projecting IL neurons and abolished social preference in a 3-chamber social test. Overall, the data suggest that the male IL is more responsive to conspecific social interaction and that increasing male IL glutamatergic activity can bidirectionally modulate social motivation, dependent upon the method of stimulation, while interneuron stimulation produces opposing effects. Additionally, the female IL has less neural activity than males after conspecific interaction and stimulation of female IL glutamate neurons does not alter sociability. Although, amygdala-projecting female IL neurons are necessary for social preference.

While the female IL may be less involved in conspecific interaction, Pereira and Morrell (2020) demonstrated that the IL plays a critical role in maternal behaviors. Using a conditioned place preference paradigm, new mother rats spend equivalent time in chambers associated with cocaine reward and pups. However, blockade of sodium conductance in the female IL leads to an exclusive preference for the cocaine-paired chamber. Furthermore, IL inactivation reduces maternal behaviors including nest building and retrievals. In fact, none of the IL-inactivated mothers fully retrieved all pups, while all vehicle-treated females did (Pereira and Morrell, 2020). Further, histological evidence indicates that the female IL shows greater activation following exposure to newborns than juvenile pups (Pose et al., 2019). Thus, the female IL may be more tuned to facilitate pup rearing than conspecific social interactions.

## Gonadal Hormone Influences

**FIGURE 1:**
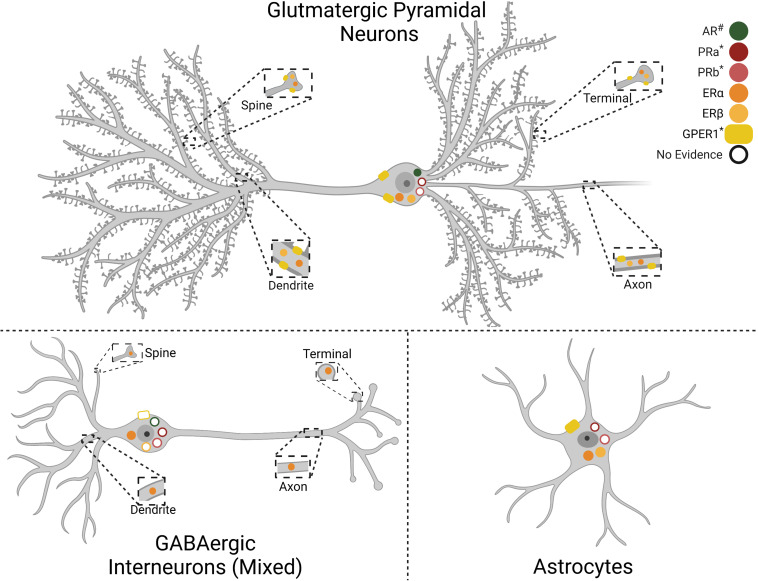
Gonadal hormone receptor expression in infralimbic cortex (IL) cell types. Depiction of gonadal hormone receptor expression and localization. Filled symbols indicate evidence for expression in the cell type and cellular compartment. Empty symbols denote lack of information as to the presence or absence of the receptor. Progesterone receptor cell-type specifity has not been reported. Androgen Receptor (AR), Progesterone Receptor A (PRa), Progesterone Receptor B (PRb), Estrogen Receptor α (ERα), Estrogen Receptor β (ERβ), G protein-coupled estrogen receptor 1 (GPER1). #AR expression is higher in males than females. *PRa, PRb, and GPER1 expression has only been reported in females. Created with BioRender.com.

The impact of gonadal hormones on neural activity may contribute to sexually divergent IL function. Gonadal hormones influence circuit regulation through both organizational effects in development as well as activational effects in adulthood. The importance of gonadal hormones for mPFC development, synapse formation, and pruning has been recently reviewed (Premachandran et al., 2020; Delevich et al., 2021). In adult rodents, estrogen receptors α and β (ERα and ERβ) are present in the male rodent mPFC (Figure 1), distributed broadly across cortical layers in both pyramidal and non-pyramidal putative interneurons (Montague et al., 2008). Further, ultrastructural analysis found ERα, ERβ, and G protein-coupled estrogen receptor 1 (GPER1) in the female mPFC. Interestingly, GPER1 is expressed at over twice the levels of ERα and ERβ, suggesting a significant fast-acting component to ER signaling (Almey et al., 2014). Moreover, ER localization is largely extranuclear, with most receptors located axonally (Almey et al., 2014). Growing evidence suggests that ER signaling plays an important role in mPFC regulation of female behavior. For instance, 17β-estradiol (E2) localized to the female PL/IL junction shifts the cognitive strategy used for maze navigation (Almey et al., 2014). Further, ERα and ERβ agonist treatment in diestrus females potentiates the antidepressant-like effect of ketamine (Dossat et al., 2018). Recent evidence from OVX females also indicates that E2 increases the excitability of IL pyramidal neurons in slice and enhances extinction of reward-seeking (Yousuf et al., 2019). While more research is needed to determine the mechanisms by which estrogens regulate prefrontal function, these studies suggest that cyclic fluctuations in intrinsic network activity impact depression-related behaviors. Considerably less is known about the effects of cortical progesterone signaling. The female rodent frontal cortex expresses both progesterone receptor a (PRa) and b (PRb), with PRb levels decreasing during estrus (Guerra-Araiza et al., 2003). Although there are no reports, to our knowledge, of PR expression in males, repeated progesterone administration increases GABA_*A*_ receptor subunit α1 expression in the mPFC of both sexes (Andrade et al., 2012). Thus, cyclic increases in progesterone likely affect mPFC E/I balance.

Androgens may also play a role in IL functional differences as androgen receptors (AR) are expressed in the frontal cortex of male and female rodents (DonCarlos et al., 2006). Expression is higher in males than females and current evidence indicates little to no astrocyte expression, though this could be age-dependent (Feng et al., 2010). Further, AR expression in midbrain-projecting neurons suggests putative pyramidal expression (Aubele and Kritzer, 2012; Low et al., 2017). In addition, androgens regulate dopamine (DA) inputs to the male rodent mPFC. Orchiectomy increases DA axonal density and extracellular DA levels within the mPFC, an effect reversed by testosterone administration (Kritzer, 2003; Aubele and Kritzer, 2012). Further, a large portion of ventral tegmental area-projecting IL neurons express AR. Taken together, these results suggest a bidirectional interaction between androgen signaling and mesocortical DA circuitry that may influence IL network excitability as well as mood and behavior. Further evidence for gonadal hormone regulation of mPFC activity comes from studies indicating that androgens and estrogens have opposing effects on mPFC metabolism of DA, norepinephrine, and serotonin during a novel environment stressor (Handa et al., 1997). Collectively, this evidence suggests gonadal hormones modify IL function. Due to the widespread expression of these receptors in mPFC cell populations and varied actions on neural activity, considerable work remains to understand how hormonal fluctuations across the lifespan impact prefrontal network function.

## Chronic Stress Impacts

The two greatest predictors of depressive outcomes are cumulative lifetime traumas and severe life stressors (Cassileth et al., 1984), indicating that the neural consequences of repeated or severe stress dictate disease burden. Mood-related symptoms, including negative affect, anhedonia, despair, and social withdrawal, are also frequently initiated and/or exacerbated by prolonged stress (Kennedy and Adolphs, 2012; Lupien et al., 2009). Accordingly, chronic stress exposure has been a primary preclinical paradigm for studying depression and mood disorders in animal models. In recent years, there has been growing interest in the sexual basis of chronic stress impacts on limbic structures. Multiple excellent reviews have covered the topic in-depth (McLaughlin et al., 2009; Bourke et al., 2012; Shansky and Woolley, 2016; Shors, 2016; Shepard and Coutellier, 2018; Fogaça and Duman, 2019; Moench et al., 2019; Page and Coutellier, 2019). Here, we review IL-specific effects.

Chronic stress-induced IL pyramidal neuron dendritic hypotrophy has been consistently reported in male rodents, though this varies based on projection targets (Cerqueira et al., 2005; Goldwater et al., 2009; Shansky et al., 2009; Luczynski et al., 2015; Czéh et al., 2018). Further, measurement of the long-term activation marker ΔFosB indicates the male IL is responsive to chronic stress exposure, an effect not present in other frontal regions such as the anterior cingulate or orbital cortices (Flak et al., 2012; Pace et al., 2020). However, studies of chronic stress effects on male IL glutamatergic excitability have yielded mixed results, contributing to opposing hypothesis of either hyper- or hypo-inhibition. McKlveen et al. (2016) found that IL pyramidal neurons of male rats exposed to a 2-week variable stress paradigm had increased inhibitory currents and more GABAergic synaptic appositions, suggesting increased inhibition of IL glutamatergic neurons. GR was also reduced specifically in PV interneurons (McKlveen et al., 2016), pointing to the importance of glucocorticoid feedback for regulating local inhibition. In support of hyper-inhibition, chemogenetic inhibition of male mouse IL PV interneurons during CVS reduces passive coping in FST (Nawreen et al., 2020). Furthermore, chronic stress increases GAD67 mRNA in the male mouse IL (Shepard et al., 2016). Additionally, 3 weeks of daily restraint stress in male mice increases dendritic arborization of GAD67-positive interneurons but reduces GAD67-positive somas (Gilabert-Juan et al., 2013). In support of hypo-inhibition, Czéh et al. (2018) found reductions in both IL interneuron populations and inhibitory currents in an anhedonic subpopulation of male rats exposed to 9 weeks of variable stress (Czéh et al., 2018). Similarly, 3 weeks of chronic unpredictable stress reduces GAD67 mRNA in male rats, although vGluT1 mRNA is also reduced (Ghosal et al., 2020). Thus, there are data to support both increased and decreased inhibition of male IL pyramidal neurons after chronic stress. Multiple factors including differences in methodology, stress paradigms, and temporal factors may contribute to the discrepant findings. Further, how these post-mortem changes affect neural network activity and behavioral outcomes remains to be determined. *In vivo*, electrophysiology studies indicate male IL neurons increase firing during a shock-predicting cue; however, this effect is not present after repeated stress (Wilber et al., 2011). Taken together, chronic stress reduces male IL glutamatergic dendritic complexity and spine density, yet the chronic stress effects on inhibitory neural populations are mixed and have yet to reach a consensus.

Numerous female studies suggest that estrogen may be protective against chronic stress effects on IL function. Though not specific to the IL, Wei et al. (2014) found that repeated restraint stress reduced mPFC miniature excitatory postsynaptic current (mEPSC) amplitude and frequency in males but not females. The decreased excitability was accompanied by a male-specific reduction in glutamate receptor surface proteins. However, ER antagonism in females unmasked stress effects on mEPSC frequency and glutamate receptors, suggesting ER prevents excitatory hypofunction following stress. Intriguingly, estrogen delivery in males is sufficient to prevent stress effects on mEPSC amplitude and frequency, as well as partially restore glutamate receptor surface protein expression (Wei et al., 2014). Moreover, female IL neurons generally do not show stress-induced dendritic remodeling. However, female IL pyramidal neurons projecting to the basolateral amygdala have estrogen-dependent increases in dendritic branching after repeated restraint stress (Shansky et al., 2010). Further, repeated stress increases spine density in this projection regardless of estrogen treatment (Shansky et al., 2010). Others have reported sex-specific effects of chronic variable stress based on IL projection target. Here, chronically-stressed female mice have increased EPSCs in the IL-NAc projection, while males have greater loss of dendritic complexity in VTA-projecting IL neurons. Additionally, chemogenetic inhibition of NAc-projecting IL neurons rescues chronic stress-induced behaviors only in females (Bittar et al., 2021). In contrast to pyramidal cells, PV interneurons appear to be more stress susceptible in females than males. Female IL PV mRNA increases following 2 weeks of daily stress exposure, with a further increase at 4 weeks. Females at 4 weeks also have increased PV neuron density and reduced IL c-Fos expression following open-field, effects that are absent in males (Shepard et al., 2016). However, both male and female mice have increased c-Fos in PV cells (Page et al., 2019), indicating increased interneuron activity. Although, chemogenetic activation of IL PV interneurons induces anxiety-like behavior only in females. Overall, these results indicate that estrogen is protective for female IL glutamatergic neurons, sex differences in chronic stress effects are projection-dependent, and interneuron populations are more susceptible to chronic stress in females.

## Conclusion

The increased attention on females in preclinical research and the rapid development of neurobiological techniques with enhanced genetic and temporal specificity have isolated sex-specific regulatory roles of IL neural populations. Manipulations that induce long-term changes in pyramidal E/I balance (SSFO-mediated hyperexcitability or lentiviral knockdown of glutamate release) can lead to divergent and sometimes contradictory behavioral outcomes. However, acutely increasing activity of IL glutamate neurons regulates numerous stress coping, motivational, and social behaviors in male rodents. The aggregate data suggest these cells promote active coping, context-appropriate reward-seeking, motivation, and sociability. Although, specific projections may have differing or even opposing actions. Less work has examined stress coping in females, but IL glutamate neurons have sexually divergent effects on physiological stress responses. Female IL pyramidal neurons may also play a smaller role in reward-seeking and motivational behaviors. In terms of sociability, the female IL seems less involved in conspecific interactions, with significant involvement in maternal behaviors. Many of these differences may be mediated by gonadal hormone signaling in different components of the IL neural and glial network. Generally, estrogens seem to protect glutamate neurons from the effects of chronic stress while androgens modulate cortical dopamine function. The sex-specific functions of the numerous IL interneuron subtypes remain to be determined. Further, the effects of chronic stress on IL cellular excitability are mixed for both sexes. Ultimately, complex interactions between sex and stress impact many aspects of vmPFC local networks and, consequently, brain-wide synaptic signaling. Determining the mechanistic basis of E/I balance in these cell groups is likely to significantly push forward our understanding of mood disorders and identify sex-specific treatment options to improve health outcomes.

## Author Contributions

Both authors contributed to the conceptualization of the review. TW performed the initial literature search, wrote the first draft of the manuscript, and created the illustrations in BioRender. BM contributed additional literature and revised the manuscript. Both authors approved the submitted version.

## Conflict of Interest

The authors declare that the research was conducted in the absence of any commercial or financial relationships that could be construed as a potential conflict of interest.

## Publisher’s Note

All claims expressed in this article are solely those of the authors and do not necessarily represent those of their affiliated organizations, or those of the publisher, the editors and the reviewers. Any product that may be evaluated in this article, or claim that may be made by its manufacturer, is not guaranteed or endorsed by the publisher.
